# Buffalo Medical and Surgical Journal—Completion of Volume Five

**Published:** 1866-07

**Authors:** 


### Editorial Department.

### Buffalo Medical and Surgical Journal—Completion of Volume Five.

Five years of editorial labor have passed away, leaving its experiences, agreeable and otherwise, for future reflection and improvement. Our journal has lived through the commercial reverses of civil war, standing alone, the only medical periodical in the State, until since the return of peace has again restored the equilibrium of trade, and allowed the sciences and arts to be cultivated under the favoring influences of political quiet and repose. It has been issued regularly, contributed to generously and ably, subscribed for extensively and paid generally in advance. It will thus be seen that the *Buffalo Medical and Surgical Journal* has much for which to be thankful and little of which to complain. With a deep sense of obligation, it has to thank the authors of original articles, transactions, correspondence, etc., etc., for their assistance in making the volume which closes with the present number, one of the most attractive and valuable of the series. We have thus been able to furnish a medical periodical, more largely original than any other similar publication in this country, and at the same time containing a *resume* of all the most important discoveries and improvements in medicine and surgery in all countries.

It must be confessed that Buffalo as a medical center, is largely undeveloped, and that our resources for observation and experience, though sufficiently large, are yet too unproductive of carefully recorded facts—of well digested, intelligently selected and reported experience. In this respect, however, we are rapidly and certaily improving; time and the wholsome stimulus of ambition will yet achieve for us a more enviable distinction. Medical observation is liable to many fallacies; so frequently are conclusions drawn upon false or inadequate grounds, that most of the recorded experience of the profession through its whole past history is now known to be largely incorrect either in aggregate or detail. Incorrect views of the nature of disease have been entertained by the profession from want of well directed investigations, from want of carefully conducted comparison and experiment.—Great advances have been made, yet no doubt much still remains to be discovered in the field of pathological observation. We have not yet learned all which may be known of the character of disease—its origin, causes, nature, tendency and termination are often undiscovered or misunderstood. Herein is a wide field for cultivation, one which will yield returns in immediate products; and which in the future will crown the honest, faithful worker, with garland wreaths of honor, as original discoverers of truth. With an ability and devotion unequaled in any other department of science the profession have directed their attention to the discovery of truth, in the nature of disease; sometimes almost seeming to forget, that such knowledge is valuable mainly when made subservient to its removal or palliation. The treatment of disease has more often received the attention of the less observing and less educated in the profession, and conclusions have been drawn upon inadequate and uncertain grounds; though it is true that the most intelligent and distinguished have also lent their names and influence, have doubtless given their confidence to useless, inert and injurious compounds, attributing to them properties which they never contain. A great many remedies have been introduced and recommended, which are of no value whatever, and which should never have been proposed, and never adopted. That the practice of medicine has undergone great change and great improvement within the past few years is the common remark. This is said with considerable self-complacency by the satisfied portion of the profession, who appear to forget that regeneration is still going on more rapidly than ever before, and that our successors will point out the fallacies and absurdities of the present, as we do the inconsistencies of the past.

These remarks are made in this connection with the view to indicate the legitimate aims and objects of medical journalism—to show what will be some of the objects in publishing the *Buffalo Medical and Surgical Journal.* If errors and mistakes are not to be pointed out, by publication of truth, then there is no longer any object in expending time, and means, upon medical periodical publications. To correct error, it is only necessary to place it in comparison with truth, it will then disappear and die; while if it were attacked by open warfare, it might gain adherents and strength, and continue to prevail.

It may be expected that we say a word of the prospects of the coming volume. We are happy again to inform our readers that it will contain such material as is furnished by the profession for publication, and that if our journal ever seems uninstructive and unentertaining, they are themselves earnestly and cordially invited to contribute to its pages the most carefully selected experience at their command—to assist in making the journal what they would have it, full of instruction and interest. We have never complained of want of support, never said that we were left to make a journal ourselves, with only the assistance of a few professional friends. Never intimated that some physicians—near neighbors of ours—read the journal regularly, and always know if it contains any sentiments disagreeable to them, but obtain it from its gratuitous or complimentary distribution, and do not appear as subscribers, paying three dollars a year, for the support of an enterprise in which they should feel an interest. We have repeatedly said that there were but few physicians in our legitimate circle, who do not cordially and generously sustain the journal; indeed we know of none; and we venture the prediction, that the world and the profession will never know them; there are a *few*, but they will never come to light.

We are happy to announce that the next volume will contain original articles, reports, translations and correspondence from the most distinguished observers, and will also contain selections from the publications of the best authors in all countries. We feel confidence that it will surpass in excellence and value any of its predecessors, since we have promise of increased assistance from our friends, interested in the progress of medical and surgical knowledge.

In conclusion, we desire to express our obligations for past favors and an earnest desire that we may merit their continuance.

